# An international study of caregiver-reported burden and quality of life in metachromatic leukodystrophy

**DOI:** 10.1186/s13023-022-02501-8

**Published:** 2022-09-02

**Authors:** Caroline Sevin, Magalie Barth, Alexandra Wilds, Abena Afriyie, Markus Walz, Annamarie Dillon, Kenneth Howie, Francis Pang

**Affiliations:** 1 Service de Neuropédiatrie, Centre de Référence des Leucodystrophies et Leucoencéphalopathies Génétiques de cause rare, CHU Paris-Sud-Hôpital de Bicêtre, Le Kremlin-Bicêtre, France; 2 Service de Génétique, Hôpital Universitaire d’Angers, Angers, France; 3 Orchard Therapeutics, 245 Hammersmith Road, London, W6 8PW UK; 4 Magnolia Innovation, Hoboken, NJ USA

**Keywords:** Metachromatic leukodystrophy, MLD, Caregiver experience, Caregiver burden, Quality of life, Burden of illness, Early-onset, Late infantile, Juvenile

## Abstract

**Background:**

Metachromatic leukodystrophy (MLD) is an autosomal recessive lysosomal disorder caused by mutations in the arylsulfatase A gene. Until now, there has been little information on the burden of MLD on patients and their caregivers. This multinational study aims to quantify caregiver-related impacts of MLD across several key domains including symptoms, treatment burden, time investment, social and emotional well-being, and professional and financial impact.

**Results:**

Data were collected through moderator-assisted web survey and telephone interviews. The survey was developed with extensive input from clinical experts and MLD patient advocacy groups. The EQ-5D-5L questionnaire was administered during follow-up interviews. The total sample consisted of parents of MLD patients in the US (*n* = 10), France (*n* = 10), Germany (*n* = 6), UK (*n* = 5), Belgium (*n* = 1), and Norway (*n* = 2). The impact of MLD is evident from the EQ-5D-5L scores, which indicate utility values for caregivers below respective national population norms and a higher proportion of caregivers reporting problems with anxiety/depression. Time involved for care was demonstrated by a mean of 4.1 inpatient and 29.6 outpatient hospital visits in the previous 12-month period. These commitments place stress on familial relationships with 50% of caregivers reporting their child’s MLD diagnosis had negatively impacted their relationship with their spouse/partner. Professionally, 76.5% of caregivers stopped working or switched to part-time employment following their child’s MLD diagnosis, and most acknowledged caring for their child had affected their potential for career progression or promotion. Differences are also observed based on late infantile versus juvenile onset MLD, time since diagnosis, and for transplanted patients versus those who received palliative care only.

**Conclusions:**

This multinational study demonstrates that MLD consistently negatively affects many aspects of caregivers’ lives including health, relationships, and professional status, irrespective of location. We expect that the results of this study are generalizable to other countries. This study enhances our understanding of MLD caregiver impacts, which could improve patient care and assist in identifying support for individuals with MLD and their families.

**Supplementary Information:**

The online version contains supplementary material available at 10.1186/s13023-022-02501-8.

## Background

Metachromatic leukodystrophy (MLD) is a rare, demyelinating lysosomal storage disorder caused by mutations in the arylsulfatase A gene. The resulting dysfunction and destruction of the central and peripheral nervous systems leads to decline and ultimately total loss in motor and cognitive functions. This relentless neurodegenerative progression is a hallmark of MLD [1, 2]. ​MLD can be clinically categorized based on age of symptom onset— the three main classifications include late infantile onset (symptom onset ≤ 30 months), juvenile onset (onset > 30 months and < 17 years) and adult onset (symptom onset ≥ 17 years) [2]. MLD has a worldwide prevalence rate of 1 in 40,000 to 160,000. Incidence is suspected to be even higher in certain populations such as among the Navajo Indian people or Arab groups of Israel [3]. Late infantile and juvenile onset MLD account for 70–90% of all MLD cases [4]. In late infantile and juvenile MLD, disease progression is rapid and aggressive, and prognosis is usually fatal [2]. Diagnosis of MLD is challenging and may take several years for some patients to receive a diagnosis; additionally, misdiagnosis is common [5, 6]. In the absence of newborn screening, pre-symptomatic patients are only identified if an older sibling was diagnosed with the disease [7]. Until recently, only palliative options existed to manage certain symptoms of the disease and in a few cases, eligible late onset patients could receive hematopoietic stem cell transplantation to stop or slow the progression of the disease; however, the benefit from transplant is minimal particularly for those who do not receive immediate care after initial onset of symptoms [8]. ​​In December 2020, a lentiviral ex vivo gene therapy was approved by the European Medicines Agency for treatment in children with late infantile or early juvenile forms who do not yet have clinical manifestations of the disease, and in children with the early juvenile form who have early manifestations of the disease [9].​

Given the debilitating nature of symptoms and limited number of effective therapeutic options, patients with MLD often require significant care. Consequently, this condition does not only affect the diagnosed individuals but can also create a substantial burden on their family caregivers. Caregivers of individuals with lysosomal storage diseases have reported multiple impacts on their lives, including personal and family relationships, personal time, daily responsibilities, physical and mental health, social life, leisure activities, work productivity, and finances [10]. However, there have been few studies which offer a multi-country perspective that is solely focused on the dynamics of caring for late infantile and juvenile onset MLD patients [6, 10]. Multi-country studies in other rare conditions highlight the significant role families play in care and the consequential burden these caregivers consistently face across countries [11–13].

This study aims to enhance our understanding of the burden of disease on family caregivers of individuals with MLD, which ultimately should improve patient care and inform the health technology assessment (HTA) of new interventions for MLD. Through caregiver-reported accounts, we capture a multi-country perspective on the holistic burden of care including physical and psychological health, time investment, and familial, social, professional and financial impacts. In addition, for the first time, we capture the quality of life impact on caregivers in the form of dis(utilities) based on the EQ-5D instrument, a generic health profile measure which also generates index values which can be used in cost-effectiveness analyses.

## Methods

### Study design and participants

A cross-sectional study was conducted in the United States (US), Germany, United Kingdom (UK), France, Belgium and Norway. Participants were primary family caregivers of at least one individual diagnosed with MLD. Caregivers of deceased MLD patients were included if the time since death did not exceed three years at the time of the study. Caregivers of MLD patients who had received gene therapy or experimental therapies in development were excluded from the study. Responses could be provided for multiple children with MLD if all inclusion and exclusion criteria were met.

### Procedures and measurements

#### Methodology

Participants were recruited through MLD patient advocacy groups and screened by researchers at Magnolia Innovation. The study sponsor was blinded to the identity of the participants. The study included a 60-min quantitative telephone-assisted web survey and a 30-min follow-up qualitative phone interview, during which the EQ-5D-5L questionnaire to measure health status was administered. Moderators administered the surveys, interviews and questionnaires in native languages for caregivers in Germany and France, respectively, and in English for all other countries.

#### Survey design and validation

This paper presents the quantitative findings from the survey and EQ-5D-5L. The content of the survey was validated by MLD clinical experts and national representatives of MLD patient advocacy groups (see list of organizations under “Declarations”). The main sections of the survey included caregiver and patient demographics, and questions on the disease management burden, time investment, and the social, emotional, psychological, and financial impacts of MLD. Where applicable, the survey captured information regarding the previous 4-week and 12-month periods. Data collection occurred over a period of approximately 12 months.

#### Measurements

Caregivers answered a series of “yes” or “no” questions and provided estimates regarding the time involved in daily caregiving duties, as well as for the stem cell transplant procedure (where appropriate). Caregivers also provided estimates for the number of times that their children with MLD had outpatient and inpatient hospital visits. Outpatient visits were defined as any medical care received that did not require an overnight hospital stay including doctor’s office, urgent care, or follow-up appointments outside the hospital setting, and inpatient hospital visits were defined as any medical care received where the child with MLD was admitted overnight.

To measure the emotional impact of providing care for a child with MLD, the caregivers were asked to select the frequency of the positive and negative emotions that they felt in the previous 4-week period, on a Likert scale with six options (all of the time, most of the time, a good bit of the time, some of the time, a little of the time, or none of the time).

The social impact of MLD was evaluated by asking the caregivers to indicate the extent to which they agreed or disagreed with a series of statements regarding their social lives since their child’s diagnosis. There were seven categories of responses: strongly disagree, disagree, somewhat disagree, neither agree nor disagree, somewhat agree, agree, or strongly agree. The caregivers were also asked how often they could keep up with their family responsibilities and social commitments in the previous 4-week period, with the following response options: never, rarely, sometimes, often, or always.

The effects of MLD on familial relationships was determined through caregiver responses to the level at which key relationships had been negatively impacted, based on the following options: not at all, little, moderate, somewhat, significantly, extremely, or not applicable.

The caregivers answered “yes” or “no” to a series of questions related to their professional lives and financial situations. Additionally, there were questions about the amount of nursing assistance received and if they paid for the services themselves.

#### Measurement consistency

While all participants were administered the same survey, the question about the hours spent as a caregiver was asked differently for caregivers in France versus the caregivers in all other countries due to differences in interpretation. In France, the caregivers were asked “How many additional hours do you spend caring for your child with MLD, outside the hours you would normally spend caregiving for a child?”, while caregivers in all other countries were asked “How many hours in total do you spend caregiving for your child with MLD?”. Therefore, these responses were evaluated separately for the caregivers in France and for caregivers in all other countries combined.

### Caregiver-reported EQ-5D-5L collection

Participants completed the EQ-5D-5L questionnaire [14] following the quantitative survey. The spouses or live-in partners of the participants were invited to complete the EQ-5D-5L questionnaire if they also provided care for the individual with MLD; however, only one caregiver was able to complete the primary caregiver questionnaire component of the study. The EQ-5D-5L measured the quality of life (health status) of caregivers across five dimensions of health: mobility, self-care, usual activities, pain/discomfort, and anxiety/depression at five levels (no problems, slight problems, moderate problems, severe problems, unable). The scores of the five dimensions were also converted to EQ-5D utility index scores for caregivers in the US, Germany, UK, and France using the EuroQol EQ-5D-5L Crosswalk Index Value Calculator [15, 16], as limited national EQ-5D-5L value sets are currently available. The utility index scores ranged from 0 to 1, where 0 indicated death and 1 indicated perfect health. The questionnaire included a visual analogue scale (VAS), which asked respondents to rate the overall current state of their health on a scale of 0 to 100, where 0 represented the worst imaginable health and 100 represented the best imaginable health. The utility index and VAS scores for caregivers in the US, Germany, UK and France were compared to the general population norms in each country [17].

Unless otherwise noted, the analysis included the responses of caregivers in the all the survey countries, namely US, Germany, UK, France, Norway and Belgium. Due to the relatively small sample of caregivers in Norway and Belgium, the responses of these caregivers were described separately only where appropriate.

## Analyses

### Subgroup analyses

Subgroup analyses aimed to evaluate outcomes for the caregivers of patients diagnosed with late infantile onset MLD compared to those diagnosed with juvenile onset MLD. Additionally, the impact of time since diagnosis, which was calculated only for the MLD patients alive at the time of interview, was evaluated by comparing outcomes of caregivers whose children had been diagnosed within the past two years (Group A) at the time of the survey, caregivers whose children had been diagnosed more than two years but less than six years (Group B) at the time of the survey, and caregivers whose children had been diagnosed six or more years ago (Group C). Lastly, outcomes were compared for the caregivers of patients who had received stem cell transplant for MLD to those who have only received palliative treatments for MLD. These subgroup analyses specifically focused on the differences in time investment, and social and familial impact.

### Statistical analyses

Descriptive statistics were used to analyze survey responses and the five dimensions of the EQ-5D-5L (mobility, self-care, usual activities, pain/discomfort, anxiety/depression). The mean, standard deviation (SD), median and range were calculated for continuous data. Proportions are reported as n (%) or percentages and due to rounding, percentages may not sum to 100 percent. The Fisher’s exact test was used to compare country-level results of the EQ-5D dimensions to the population norms in each country, as well as compare the social and familial impact outcomes for the caregivers of individuals diagnosed with late infantile MLD to the caregivers of individuals diagnosed with juvenile MLD. The one-sample Wilcoxon signed rank test was used to compare country-level EQ-5D utility index and VAS scores to the population norms in each country. The time investment outcomes for the caregivers of individuals who had received stem cell transplant were compared to those of the caregivers of individuals who had only received supportive care using the Mann–Whitney U test. The Fisher-Freeman-Halton test was used to compare the social impact outcomes for the three groups of caregivers stratified by the time since child’s MLD diagnosis, and the Kruskal–Wallis test was used compare the time investment outcomes between the groups. Analyses were conducted using SPSS version 28. A *p value* (two-sided) of less than 0.05 was considered statistically significant.

## Results

### Caregiver and patient characteristics

A total of 34 participants completed the quantitative survey. Ten caregivers lived in the United States and 24 lived in Europe (Germany = 6, United Kingdom = 5, France = 10, Norway = 2, Belgium = 1). The characteristics of the total sample and their children with MLD, as well as country-level data for the US, Germany, UK and France are summarized in Table 1.

**Table 1:** Caregiver and patient demographics

		Total (*N* = 34)	US (*n* = 10)	Germany (*n* = 6)	UK (*n* = 5)	FR (*n* = 10)
Caregivers						
	Gender, female	28 (82.4)	8 (80)	4 (66.7)	5 (100)	9 (90)
	*Age*					
	20–29 years	5 (14.7)	1 (10)	1 (16.7)	1 (20)	1 (10)
	30–39 years	11 (32.4)	5 (50)	1 (16.7)	2 (40)	3 (30)
	40–49 years	11 (32.4)	2 (20)	2 (33.3)	2 (40)	4 (40)
	50–59 years	5 (14.7)	1 (10)	2 (33.3)	0 (0)	1 (10)
	60–69 years	2 (5.9)	1 (10)	0 (0)	0 (0)	1 (10)
	*Relationship status*					
	Married	25 (73.5)	1 (10)	6 (100)	2 (40)	7 (70)
	Living with a partner	8 (23.5)	7 (70)	0 (0)	3 (60)	3 (30)
	Single	1 (2.9)	2 (20)	0 (0)	0 (0)	0 (0)
	*Employment status*					
	Full-time	8 (23.5)	4 (40)	2 (33.3)	1 (20)	0 (0)
	Part-time	12 (35.3)	3 (30)	2 (33.3)	0 (0)	6 (60)
	Unemployed	14 (41.2)	3 (30)	2 (33.3)	4 (80)	4 (40)
	Household income*					
	< 25,000	4 (12.5)	1 (10)	0 (0)	2 (40)	1 (10)
	25,000–49,999	11 (34.4)	4 (40)	0 (0)	1 (20)	6 (60)
	50,000–74,999	7 (21.9)	1 (10)	2 (50)	1 (20)	2 (20)
	75,000–99,999	6 (18.8)	2 (20)	2 (50)	0 (0)	1 (10)
	100,000–149,999	4 (12.5)	2 (20)	0 (0)	1 (20)	0 (0)
MLD Patients						
	Number of MLD Patients	35	10	6	5	11
	Gender, female	22 (62.9)	5 (50)	5 (83.3)	5 (100)	6 (54.5)
	Age (years)**					
	Mean (SD)	9.9 (7.5)	10.2 (8.4)	7.8 (6.2)	9.0 (4.4)	9.1 (5.9)
	Median (range)	8.0 (2.3 – 33.3)	8.3 (3 – 30.3)	4.6 (3.2 – 18.4)	6.8 (4.6 – 15.4)	9.0 (2.3 – 17.0)
	Time since MLD Diagnosis (years)**					
	Mean (SD)	5.1 (4.4)	5.1 (4.8)	2.8 (1.9)	4.9 (2.9)	5.1 (4.7)
	Median (range)	3.3 (0.3 – 16.1)	3.2 (0.8- 16.1)	2.5 (0.8 -5.9)	3.9 (1.9- 9.3)	4.3 (0.3- 12.5)
	*Time of symptom onset*					
	Late infantile (≤ 30 months old)	21 (60)	6 (60)	4 (66.7)	3 (60)	7 (63.6)
	Juvenile (between 30 months and 17 years old)	13 (37.1)	3 (30)	2 (33.3)	2 (40)	4 (36.4)
	Borderline late infantile and juvenile#	1 (2.9)	1 (10)	0 (0)	0 (0)	0 (0)

*Currencies were USD for US, Euros for Germany, France, Pound Sterling for UK; 2 Germany respondents elected not to share income; **4 deceased individuals with MLD were excluded from calculation; #One individual with MLD was diagnosed by a physician as an unusual case of borderline late infantile and juvenile MLD

### Caregiver respondent characteristics

All caregivers were parents, mostly mothers (82.4%), of the individuals with MLD. Each caregiver in the US, UK, Norway and Belgium, seven of the ten caregivers in France (70.0%), and five of the six caregivers in Germany (83.3%) reported on one living child with MLD. Two caregivers in France (20.0%) and one caregiver in Germany (16.7%) reported on one child with MLD who had passed away. One caregiver in France (10.0%) reported both on one living child with MLD and one child with MLD who had passed away. In total, the caregivers reported on 31 living children and four deceased children for a total of 35 individuals with MLD within the study sample.

Most of the caregivers were in the 30- to 40-year-old age groups (64.8%) and were married or living with a partner (97.0%) at the time of the study. The respondents were also predominately not currently employed or working part-time (76.5%).

### MLD patient sample characteristics

Most of the individuals with MLD (22/35, 62.9%) were female. The mean age of the 31 living individuals was 9.9 (SD = 7.5) years and the mean time since diagnosis with MLD was 5.1 (SD = 4.4) years. Most of the individuals with MLD (*n* = 35) were diagnosed with late infantile onset MLD (60.0%) and juvenile onset MLD (37.1%). One individual with MLD (2.9%) could not be categorized due to an unusual diagnosis as a borderline late infantile and juvenile MLD. Two individuals with MLD (5.7%) were diagnosed through genetic testing as a result of a sibling’s diagnosis, while the rest were diagnosed symptomatically.

Of the individuals with MLD who were alive at the time of interview (*n* = 31), all but two were at advanced stages of their disease (i.e., severe cognitive impairment and loss of trunk control). Of the individuals with late infantile MLD, majority required a feeding tube (19/21, 90.5%) and use of a wheelchair (18/21, 85.7%). Individuals with juvenile MLD were also often tube fed (6/13, 46.2%) and wheelchair-bound (11/13, 84.6%). Additionally, of those who were alive at the time of interview (*n* = 18 late infantile and 12 juvenile individuals), most caregivers reported their child having trouble with speech, experiencing seizures, and being unable to use the toilet independently in the previous 4-week period. Of the individuals with late infantile MLD, all either had trouble with speech or were unable to communicate in the previous 4-week period. All late infantile cases were also unable to use the toilet on their own based on the same time period. Seizures (15/18, 83.3%) were also reported by most caregivers for their child with late infantile MLD in the past 4-week period. Caregivers also reported that most individuals with juvenile MLD either had trouble with speech or were unable to speak (10/12, 83.3%) and experienced seizures (6/12, 50.0%) in the previous 4-week period. None of the juvenile MLD individuals were able to use the toilet fully on their own in the past 4-week period. Lastly, six individuals with MLD (three in the US, and one each in Germany, France and Belgium) had received stem-cell transplant for MLD (6/35, 17.1%), and the remaining individuals with MLD (82.9%) had only received palliative care.

### EQ-5D-5L dimension scores

Eight of the 10 caregivers in the US, and all the caregivers in Germany (*n* = 6), the UK (*n* = 5), France (*n* = 10) and Norway (*n* = 2) completed the EQ-5D-5L questionnaire. Additionally, one caregiver’s spouse in Germany, one in the UK, and one in Norway also completed the EQ-5D-5L questionnaire. The caregiver in Belgium did not complete the questionnaire. Of the 34 respondents that completed the questionnaire, three (8.8%) reported difficulties with mobility, one (2.9%) reported difficulties with self-care, 14 (41.1%) reported difficulties doing usual activities, 22 (64.7%) were experiencing pain or discomfort, and 23 (67.6%) were experiencing anxiety or depression (see Additional file 1: Sect. 1 for caregiver quotes on psychological and physical burdens of MLD). Country-level results are described below and in Table 2.

**Table 2:** Caregiver ED-D5-5L Dimension Scores: Proportion Reporting Any Problems

	US (*n* = 8)	Germany (*n* = 7)	UK (*n* = 6)	France (*n* = 10)
*Mobility*	0%	14.3%	16.7%	0%
Population norm	18.5%	15.9%	18.4%	13.4%
*Self-care*	0%	0%	16.7%	0%
Population norm	3.7%	2.7%	4.3%	4%
*Usual activities*	37.5%	71.4%	33.3%	30%
Population norm	17.9%	9.9%	16.3%	10%
		*(p* < *0.001)*		
*Pain/discomfort*	75%	42.9%	66.7%	70%
Population norm	48.3%	27.6%	33%	35.9%
				*(p* = *0.042)*
*Anxiety/depression*	75%	57.1%	83.3%	80%
Population norm	23.2%	4.3%	21%	15%
		*(p* < *0.001)*	*(p* = *0.002)*	*(p* < *0.001)*

### United States EQ-5D-5L dimension scores

In the US, the proportion of caregivers who reported difficulties doing usual activities and were experiencing pain or discomfort and anxiety or depression were directionally higher than the general US population norms*.* The proportions of caregivers who reported difficulties with mobility and self-care were lower than the general US population norms. These differences were not statistically significant.

### Germany EQ-5D-5L dimension scores

Among the caregivers in Germany, the proportion that reported difficulties doing usual activities and those who were experiencing anxiety or depression were significantly higher than the general population norms in the country *(p* < *0.001)*. The proportion of caregivers reporting problems with pain or discomfort was higher than the general population norm, but the differences were not statistically significant.

### United Kingdom EQ-5D-5L dimension scores

The proportion of caregivers who were experiencing anxiety or depression was significantly higher than that of the general population norm in the UK *(p* < *0.001)*. The percentages of caregivers who reported that they had difficulties with self-care and problems doing usual activities were also higher than the country’s population norms, although these differences were not statistically significant.

### France EQ-5D-5L dimension scores

Compared to the general population norms in France, the percentages of caregivers who were experiencing pain or discomfort *(p* = *0.042)* and anxiety or depression *(p* < *0.001)* were significantly higher. The proportion of caregivers who reported problems doing usual activities was also higher than that of the general population, but these differences were not statistically significant.

### Norway EQ-5D-5L dimension scores

The two caregivers in Norway both reported no difficulties with self-care and doing usual activities and were not experiencing anxiety or depression at the time of the survey. One had no problems with mobility and was not experiencing pain or discomfort, while the other reported having problems in both of these domains.

### Caregiver EQ-5D-5L utility index scores

**Fig. 1:**
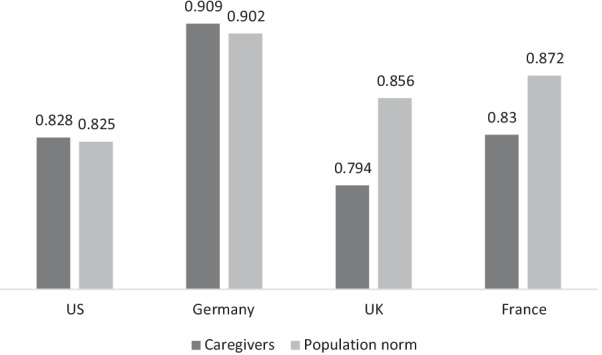
Median EQ-5D Utility Index Scores of Caregivers and Population Norms. US caregivers versus population norm: *p* = 0.575; Germany caregivers versus population norm: *p* = 0.495; UK. caregivers versus population norm: *p* = 0.116; France caregivers versus population norm: *p* = 0.332

The median index scores among the caregivers in the US and Germany were slightly higher compared to the population norms of each respective country, while the median scores in the UK and France were lower compared to the population norms of each respective country (Fig. 1). However, none of these differences were statistically significant. Individual index scores could not be calculated for the caregivers in Norway because the crosswalk value sets for the most recent 5 level extension of the EQ-5D- instrument that are used to generate the index scores were not available for Norway.

### Caregiver EQ-5D-5L VAS scores

There were no statistically significant differences in the median VAS scores for caregivers in the US, Germany, and France compared to population norms of the respective counties.

## Time investment

### Outpatient and inpatient visits and hospital stays

Results for the total sample (*n* = 31) and country-level data are shown in Table 3. Data were not included for caregivers whose children with MLD had passed away prior to study. Overall, caregivers reported an average of 29.6 outpatient visits for their children living with MLD in the previous 12-month period. Caregivers reported an average of 2.8 outpatient visits in the previous 4-week period, with the reported number of visits ranging from 0 to 16 (see Additional file 1: Sect. 2, Quote 1 for caregiver quote on healthcare visits).

**Table 3:** Hospital visits and hospital stays for MLD patients

	Total (*N* = 31)	US (*n* = 10)	Germany (*n* = 5)	UK (*n* = 5)	France (*n* = 8)
*Number of outpatient visits in the previous 12 months*					
Mean (SD)	29.6 (41.7)	30.2 (26.1)	5.8 (3.8)	16.8 (17.6)	62.5 (65. 6)
Median (range)	15.0 (0 – 200)	22.0 (0 – 90.0)	5.0 (1.0 – 11.0)	12.0 (0 – 40.0)	43.0 (5.0 – 200)
*Number of outpatient visits in the previous 4 weeks*					
Mean (SD)	2.8 (4.2)	3.2 (3.5)	0.6 (0.5)	1.2 (1.6)	5.8 (6.3)
Median (range)	1.0 (0 – 16)	2.5 (0 – 12.0)	1.0 (0 – 1.0)	1.0 (0 – 4.0)	2.0 (1.0 – 16.0)
*Number of inpatient hospital visits in the previous 12 months*					
Mean (SD)	4.1 (9.7)	2.9 (3.0)	2.4 (2.3)	2.2 (1.8)	7.8 (19.1)
Median (range)	2.0 (0 – 55)	2.0 (0 – 9)	2.0 (0 – 6.0)	2.0 (0 – 5.0)	0.5 (0 – 55.0)
*Number of inpatient hospital visits in the previous 4 weeks*					
Mean (SD)	0.6 (1.0)	0.7 (0.9)	0.4 (0.5)	0.6 (0.9)	0.6 (1.4)
Median (range)	0 (0 – 4)	0.5 (0 – 3.0)	0 (0 – 1.0)	0 (0 – 2.0)	0 (0- 4.0)
*Inpatient hospital stays (days) in the previous 12 months*					
Mean (SD)	11.8 (18.3)	10.7 (10.8)	10.8 (14.1)	12.0 (10.5)	15.9 (32.7)
Median (range)	5.0 (0 – 95)	9.5 (0 – 33.0)	5.0 (0 – 35.0)	10.0 (0 – 28.0)	1.5 (0 – 95.0)
*Inpatient hospital stays (days) in the previous 4 weeks*					
Mean (SD)	1.6 (3.7)	3.8 (5.8)	1.0 (1.7)	0.6 (0.9)	0.6 (1.4)
Median (range)	0 (0 – 14)	0.5 (0 – 14.0)	0 (0 – 4.0)	0 (0 – 2.0)	0 (0- 4.0)

The average number of inpatient hospital visits reported by caregivers for the previous 12-month period was 4.1. Some caregivers (8/31, 25.8%) reported that their child with MLD did not receive inpatient care in the previous 12-month period, while others (5/31, 16.1%) reported over five inpatient hospital visits. For each inpatient hospital visit in the previous 12-month period, the caregivers reported that the average length of stay was 11.8 days. The number of days spent at the hospital ranged from no overnight stay to 95 days. For the previous 4-week period, caregivers reported an average number of inpatient visits that was less than one and ranged from 0 to 4 visits. For each of these inpatient hospital visits in the previous 4-week period, the individuals with MLD were admitted for a mean of 1.6 days.

### Outpatient and inpatient visits and hospital stays by time since diagnosis

Caregivers in Group B (individuals with MLD diagnosed more than two years but less than six years) reported the lowest average number of outpatient visits in the previous 12-month and 4-week periods, while caregivers in Group A (diagnosed within two years) had the highest average number of outpatient visits during both time periods. There was a statistically significant difference between the groups for the 4-week period, but not for the 12-month period. Caregivers in Group A also reported the highest number of inpatient hospital visits and highest average number of days spent in the hospital for each hospitalization for the previous 12-month and 4-week periods. For the number of inpatient hospital visits, there was a statistically significant difference between the groups for the 12-month period, but not for the 4-week period. In comparing the number of days spent at the hospital, there was statistically significant difference between the groups for both time periods. The results are shown in Table 4.

**Table 4:** Hospital Visits and Hospital Stays by Time Since Diagnosis

	Group A (*n* = 10)	Group B (*n* = 11)	Group C (*n* = 10)	*P*-value*
*Number of outpatient visits in the previous 12 months*				
Mean (SD)	56.4 (62.9)	12.6 (12.7)	21.6 (21.1)	
Median (range)	38.0 (0 – 200)	8.0 (1 – 40)	16.0 (0 – 65)	0.157
*Number of outpatient visits in the previous 4 weeks*				
Mean (SD)	6.1 (6.1)	0.6 (0.9)	1.9 (1.7)	
Median (range)	3.0 (0 – 16)	0.0 (0 – 3)	1.5 (0 – 5)	0.009
*Number of inpatient hospital visits in the previous 12 months*				
Mean (SD)	9.1 (16.3)	2.0 (1.8)	1.3 (2.0)	
Median (range)	3.5 (1 – 55)	2.0 (0 – 5)	0.5 (0 – 6)	0.012
*Number of inpatient hospital visits in the previous 4 weeks*				
Mean (SD)	1.1 (1.4)	0.5 (0.7)	0.3 (0.7)	
Median (range)	1.0 (0 – 4)	0.0 (0 – 2)	0.0 (0 – 2)	0.192
*Inpatient hospital stays (days) in the previous 12 months*				
Mean (SD)	24.2 (27.2)	6.7 (8.4)	4.9 (6.8)	
Median (range)	17.5 (3 – 95)	5.0 (0 – 28)	1.0 (0 – 15)	0.011
*Inpatient hospital stays (days) in the previous 4 weeks*				
Mean (SD)	4.2 (5.6)	0.7 (1.3)	0.1 (0.3)	
Median (range)	1.5 (0 – 14)	0.0 (0 – 4)	0.0 (0 – 1)	0.033

*Group A*: Caregivers of individuals with MLD who were diagnosed within the past ≤ 2 years; *Group B*: Caregivers of individuals with MLD who were > 2 and ≤ 6 years since diagnosis; *Group C*: Caregivers of individuals with MLD who were > 6 years since diagnosis; *P-value between groups A, B, C

Few caregivers (3/31, 9.7%) reported that their child with MLD did not receive any care at an outpatient facility during the previous 12-month period, while some (6/31, 19.4%) reported visiting outpatient facilities over 50 times. The median time since MLD diagnosis was 9.1 years for the children of caregivers reporting zero outpatient visits in the past 12-month period and 0.8 years for the children of those reporting over 50 outpatient visits in the same time period. However, the difference in time since diagnosis between the groups was not statistically significant (*p* = 0.053).

### Outpatient and inpatient visits and hospital stays by treatment received

For the previous 12-month and 4-week time periods, caregivers of individuals who had received stem cell transplant for treatment of MLD (*n* = 5) reported lower average numbers of outpatient and inpatient hospital visits and a lower average number of days spent in the hospital for each inpatient visit compared to the caregivers of individuals who had only received supportive care (*n* = 26). However, the differences observed across the two groups were not statistically significant. The results are shown in Table 5.

**Table 5:** Hospital visits and hospital stays by treatment received

	Stem cell transplant (*n* = 5)	Supportive care only (*n* = 26)	*P*-value
*Number of outpatient visits in the previous 12 months*			
Mean (SD)	14.8 (15.1)	32.5 (44.7)	
Median (range)	11.0 (0 – 40)	17.5 (0 – 200)	0.514
*Number of outpatient visits in the previous 4 weeks*			
Mean (SD)	1.8 (2.2)	3.0 (4.5)	
Median (range)	1.0 (0 – 5)	1.0 (0 – 16)	0.775
*Number of inpatient hospital visits in the previous 12 months*			
Mean (SD)	2.6 (1.7)	4.3 (10.6)	
Median (range)	3.0 (1 – 5)	2.0 (0 – 55)	0.620
*Number of inpatient hospital visits in the previous 4 weeks*			
Mean (SD)	0.4 (0.6)	0.7 (1.1)	
Median (range)	0.0 (0 – 1)	0.0 (0 – 4)	0.897
*Inpatient hospital stays (days) in the previous 12 months*			
Mean (SD)	5.6 (5.6)	13.0 (19.7)	
Median (range)	5.0 (0 – 15)	8.0 (0 – 95)	0.658
*Inpatient hospital stays (days) in the previous 4 weeks*			
Mean (SD)	0.4 (0.6)	1.9 (4.0)	
Median (range)	0.0 (0 – 1)	0.0 (0 – 14)	0.856

### Time spent acting as a caregiver

Caregivers in France (*n* = 7) reported that in the previous 12-month period, they spent an average of 5.7 (SD = 2.6) additional hours per day providing care to their child due to their MLD, outside of the usual number of hours they would spend caring for their child. For all other countries, caregivers (*n* = 23) reported that in the previous 12-month period, they spent an average of 15.2 (SD = 6.7) hours per day providing care to their child with MLD. The means and medians for the two sets of data were similar (see Additional file 1: Sect. 2, Quote 2 for caregiver quote on time involved in care).

## Familial impact

Table 6 shows the proportion of caregivers who indicated that familial relationships were negatively impacted moderately, somewhat, significantly or extremely due caregiving for a child with MLD. Half of the caregivers reported that the relationship with their spouse or partner was negatively impacted relationship, while a much fewer number (3/34, 8.7%) reported that their relationship with their child with MLD had been negatively impacted. Overall, a large proportion of caregivers (27/34, 79.4%) reported that a familial relationship had been negatively impacted by MLD (see Additional file 1: Sect. 3 for caregiver quote on familial impact).

**Table 6:** Proportion of caregivers who reported negatively impacted relationships

	Total (*N* = 34)	Late infantile MLD (*n* = 20)	Juvenile MLD (*n* = 13)	Group A (*n* = 10)	Group B (*n* = 11)	Group C (*n* = 10)
With Spouse/Partner	17 (50)	9 (45)	7 (53.9)	3 (30)	7 (63.7)	5 (50)
Between Your Children	13 (38.2)	8 (40)	5 (38.5)	3 (30)	2 (18.2)	5 (50)
With Other Immediate Family Members	14 (41.1)	8 (40)	6 (46.2)	3 (30)	6 (54.6)	4 (40)
With Your Other Children	13 (38.2)	9 (45)	3 (23.1)	2 (20)	5 (45.5)	4 (40)
With Your Child	3 (8.7)	2 (10)	1 (7.7)	0 (0)	2 (18.2)	1 (10)
Any familial relationship	27 (79.4)	16 (80)	10 (76.9)*	7 (70)	9 (81.8)	8 (80)**

Results represent caregivers who selected “moderate”, “somewhat”, “significant” or “extremely” negative impact; Caregiver of child with borderline late infantile/juvenile MLD (*n* = 1) was not included in the late infantile versus juvenile analysis; Caregivers who only had one deceased child (*n* = 3) were not included in the Group A-C analysis; Group A: Caregivers of individuals with MLD who were diagnosed within the past ≤ 2 years; Group B: Caregivers of individuals with MLD who were > 2 and ≤ 6 years since diagnosis; Group C: Caregivers of individuals with MLD who were > 6 years since diagnosis; *P-value between the late infantile and juvenile groups = 1.000; **P-value between groups A, B, C = 0.873.

### Familial impact subgroup analysis

Compared to caregivers whose children had been diagnosed with juvenile MLD, a slightly higher percentage of caregivers whose children who had been diagnosed with late infantile MLD reported that a familial relationship had been negatively impacted by the disease. The difference between the two groups, however, was not statistically significant *(p* = *1.000).* When caregivers of children who had been diagnosed with MLD in the previous two years (Group A), were compared to those whose children had been diagnosed over two years but less than six years (Group B), and those whose children had been diagnosed within six or more years (Group C), Group A had the lowest proportion of caregivers reporting that a familial relationship had been negatively impacted by their child’s diagnosis. However, there was not a statistically significant difference between the three groups *(p* = *0.873)*.

## Social impact

Results are depicted in Table 7. Of the 34 caregivers, most had to make significant lifestyle changes following their child’s MLD diagnosis (94.1%), were no longer as socially active (82.4%), and missed many of their leisure activities (55.9%). Most caregivers (61.8%) also reported some extent of dissatisfaction with their personal lives. However, majority (58.8%) reported that in the previous 4-week period, they were always or often able to keep up with their family responsibilities and social commitments (see Additional file 1: Sect. 4 for caregiver quote on social impact).

**Table 7:** Social impact of MLD

	Total (*N* = 34)	Late infantile MLD (*n* = 20)	Juvenile MLD (*n* = 13)	*P* -value†	Group A (*n* = 10)	Group B (*n* = 11)	Group C (*n* = 10)	*P*-value††
Changes in social life*								
Just As Active Socially	6 (17.6)	3 (15)	3 (23.1)	0.659	2 (20)	2 (18.2)	2 (20)	1.000
Miss Many Leisure Activities	19 (55.9)	10 (50)	8 (61.6)	0.722	4 (40)	8 (72.7)	5 (50)	0.357
Made Significant Lifestyle Changes	32 (94.1)	19 (95)	12 (92.3)	1.000	9 (90)	11 (100)	9 (90)	0.527
*Satisfaction with personal life*								
Not at all	4 (11.8)	2 (10)	2 (15.4)		2 (20)	2 (18.2)	0 (0)	
A little	8 (23.5)	5 (25)	3 (23.1)		2 (20)	2 (18.2)	2 (20)	
Somewhat	9 (26.5)	6 (30)	3 (23.1)		3 (30)	1 (9.1)	4 (40)	
Quite	7 (20.6)	3 (15)	4 (30.8)		3 (30)	1 (9.1)	3 (30)	
Very	6 (17.6)	4 (20)	1 (7.7)		0 (0)	5 (45.5)	1 (10)	
Extremely	0 (0)	0 (0)	0 (0)		0 (0)	0 (0)	0 (0)	
Dissatisfied with personal life**	21 (61.8)	13 (65)	8 (61.6)	1.000	7 (70)	5 (45.5)	6 (60)	0.596
*Ability to keep up with responsibilities and commitments*								
Always	8 (23.5)	3 (15)	5 (38.5)		2 (20)	2 (18.2)	2 (20)	
Often	12 (35.3)	7 (35)	5 (38.5)		3 (30)	4 (36.4)	5 (50)	
Sometimes	10 (29.4)	7 (35)	2 (15.4)		4 (40)	3 (27.3)	2 (20)	
Rarely	4 (11.8)	3 (15)	1 (7.7)		1 (10)	2 (18.2)	1 (10)	
Never	0 (0)	0 (0)	0 (0)		0 (0)	0 (0)	0 (0)	
Usually#	20 (58.8)	10 (50)	10 (76.9)	0.159	5 (50)	6 (54.6)	7 (70)	0.732

*Results represent caregivers who selected “agree” or “strongly agree” to social life change since child’s MLD diagnosis; **Sum of “not at all”, “a little” and “somewhat” satisfied with personal life; # Sum of “always” and “often” able to keep up with family responsibilities and social commitments in the previous 4-week period; Caregiver of child with borderline late infantile/juvenile MLD (*n* = 1) was not included in the late infantile versus juvenile analysis; Caregivers who only had one deceased child (*n* = 3) were not included in the Group A-C analysis; Group A: Group A: Caregivers of individuals with MLD who were diagnosed within the past ≤ 2 years; Group B: Caregivers of individuals with MLD who were > 2 and ≤ 6 years since diagnosis; Group C: Caregivers of individuals with MLD who were > 6 years since diagnosis; †*P*-value between late infantile and juvenile groups; ††*P*-value between groups A, B, C

### Social impact subgroup analysis

Compared to caregivers whose children were diagnosed with juvenile MLD, a slightly higher proportion of caregivers whose children were diagnosed with late infantile MLD had to make significant lifestyle changes following the MLD diagnosis *(p* = *1.000)* and reported dissatisfaction with their personal lives *(p* = *1.000)*. A higher proportion of the juvenile MLD group was usually able to keep up with family responsibilities and social commitments in the previous 4-week period compared to the late infantile group *(p* = *0.159)*.

In comparing Groups A, B and C, a similar proportion (9/10, 90%) of caregivers in Groups A and C made significant lifestyle changes following their child’s MLD diagnosis, while the proportion for Group B was the highest (11/11, 100%). Caregivers in Group A were the most dissatisfied with their personal lives while those in Group B were the least dissatisfied with their personal lives. Compared to Groups A and B, Group C had the highest proportion of caregivers who were usually able to keep up with their family responsibilities and social commitments in the previous 4-week period. However, there were no statistically significant differences between the three groups with regard to lifestyle changes *(p* = *0.527)*, dissatisfaction with personal lives *(p* = *0.596)* or the ability to keep up with responsibilities and commitments *(p* = *0.732)*.

## Emotional impact

Almost half of the caregivers reported that they felt impaired by worry for the future (16/34, 47.1%) or overwhelmed (16/34, 47.1%) or isolated (15/34, 44%) either a good bit of the time, most of the time, or all of the time in the previous 4-week period. Fewer caregivers (13/34, 38.3%) felt ‘downhearted and blue’ either a good bit of the time, most of the time, or all of the time during the same time period (see Additional file 1: Sect. 5 for caregiver quote on emotional impact).

Over half of the caregivers (18/34, 53.0%) reported that they felt happy either a good bit of the time, most of the time, or all of the time during the same time period. However, much fewer caregivers felt calm and peaceful (10/34, 29.4%) or energetic (6/34, 17.6%) either a good bit of the time, most of the time, or all of the time during the same time period. Most caregivers (24/34, 70.6%) reported that during this period, their emotional state slightly or moderately interfered with your social activities with family, friends, neighbors, or groups.

## Professional and financial impact

### Professional impact

All caregivers who were unemployed or employed part-time (26/34, 76.5%) reported that their employment conditions were a result of their decision to provide care for their child with MLD. Regardless of employment status, most caregivers (28/34, 82.4%) reported that their employment status had been affected due to caring for a child with MLD (see Additional file 1: Sect. 6 for caregiver quote on professional impact).

A large proportion of the caregivers (23/34, 67.6%) believed that their potential for promotion or career progression had been affected due to caring for a child with MLD. Of the caregivers who were employed full-time or part-time (n = 20), 40.0% reported that they experienced work problems or difficulties during the previous 4-week period and 95.0% reported that they had accomplished less than they would have liked to in the same period. Twenty-four caregivers provided a response when asked if they or their spouse or partner had missed work in the previous 4-week period due to their child’s MLD. The average number of missed workdays in the previous 4-week period as a result of a child’s MLD was 3.6 (SD = 6.4) and the number of missed workdays ranged from 0 to 29 days. The caregivers (*n* = 23) reported that on average, 45.2% of the missed workdays were unpaid.

### Nursing assistance

Most caregivers (70.5%) had received nursing assistance at some point since their child’s MLD diagnosis. Of these caregivers (*n* = 24), 70.8% (17/24) had received in-home nursing assistance and 47.8% (11/23) had incurred out-of-pocket costs for the assistance received. Caregivers (*n* = 21) reported that in the previous 4-week period, they received on average, 5.2 (SD = 5.4) hours of nursing assistance per day, and the hours of assistance ranged from 0 to 16 per week.

## Impact of transplant procedure

Of the six caregivers whose children received stem cell transplant, 5 (83.3%) relocated from their homes to stay near transplant center during the treatment. Five caregivers (83.3%) also missed work due to the transplant procedure. Four caregivers (66.7%) had to make childcare arrangements for their other children while their children with MLD were receiving transplant.

## Discussion

The purpose of this study was to capture the burden of MLD on family members and the impacts of caregiving on overall health, time, relationships, social and emotional well-being, professional status and familial finances. Very few studies have assessed the effects of MLD on the family caregivers [6, 10, 18, 19], and this study adds to the current literature by providing results for multiple countries and investigating the effects of the time since MLD diagnosis, treatments received, and onset type on caregiver quality of life. When looking at other multi-country caregiver-reported burden of disease studies, there is limited cross-continental data available. Furthermore, few studies use validated tools, such as the EQ-5D-5L and offer comparisons to national population norms [11–13]. The multi-country data in this study adds to the broader international evidence on impact of rare diseases by illustrating life-altering effects on caregivers across several domains.

### Direct health impact

The country-level results of the EQ-5D-5L suggests that family caregivers of individuals with MLD may disproportionality suffer both mentally due to anxiety or depression, and physically due to pain or discomfort. These findings are consistent with those of previous research [6, 18], which assessed the quality of life of caregivers and families of individuals with MLD. The psychological effects of providing care to a child with MLD may stem from guilt, grief, or feelings of helplessness as caregivers are confronted with the relentless and progressive physical and mental decline and imminent death of their child [6]. Caregivers may experience pain or discomfort resulting from daily physical activities related to caregiving such as lifting or transporting an immobile child from bed to a wheelchair [19]. Particularly in countries where significant differences were observed compared to population norms (i.e., Germany, UK, and France), there may be an opportunity to better connect families with the support services that will help them meet the physical demands as well as psychological impacts of caring for a child with MLD.

### Time investment

Our data highlights the extensive amount of time involved in providing care for a child with MLD. Respondents dedicate the vast majority of their time caring for their child with MLD. In addition to the time spent on outpatient and hospital visits, caregiving may also involve addressing activities of daily living such as bathing, dressing, or feeding the child with MLD [19], and tending to the medical needs of their child, which could include administering medications and frequently repositioning of a bed-ridden child [20]. These activities together can consume nearly two-thirds or more of a caregiver’s day, or six additional hours of care outside of the normal responsibilities of caring for a child. In regard to the time spent on visits to the hospital, similar results have been observed in previous research, where caregivers reported frequent trips each month to receive care from primary care physicians and specialists [6]. Our study showed that caregivers on average report a similar amount of time providing care to their child with MLD, even when there is an in-home nurse available. This finding may demonstrate how the ability to have nursing assistance often does not change caregivers’ perceptions of the time-consuming nature of MLD and that nursing assistance is supplementary not substitutive. Future research is needed to further explore the dynamic between family caregivers and the in-home nurses who also provide care for the child with MLD, and evaluate caregiver experiences with nursing care such as finding the appropriate caregiver for their child with MLD.

### Impact on familial relationships

Most respondents (79.4%) in our study reported some extent of strain on familial relationships, especially the relationships with their spouses or partners. These negative effects could be attributable to any of the factors evaluated in this research, such as the remarkable amount of time that is spent providing care, the mental or physical health effects of providing care, or the changes to a caregiver’s professional status, family income or social activities. The impact of MLD on familial relationships have been reported in previous research [6, 10]—twenty percent of caregivers in the Eichler, et al., 2016 study reported that they experienced relationship difficulties with their spouse [6], while in our study, 50% of caregivers reported that their relationship with their spouse or partner had been negatively impacted as a result of providing care for their child with MLD.

Over a third of caregivers in our study also reported a negative impact on their relationship with their other children (not diagnosed with MLD) —another consequence of the amount of attentive care required for MLD, leaving limited time devoted to their other children. Additional research could explore the impact of MLD on other children in the family as this phenomenon has not been well-explored in the literature.

Furthermore, there are families with multiple children diagnosed with MLD [7, 21]. This could lead to greater burden on familial relationships, particularly for the familial caregiver. Research could explore the burden of providing care to multiple children with MLD.

### Impact on lifestyle and career

Almost all caregivers in our research made significant lifestyle changes following their child’s MLD diagnosis and most reported some extent of dissatisfaction with their personal lives. Similar findings were reported by Harrington, where all study respondents reported that caregiving for a child with MLD had impacted their social lives [10]. These changes may not only be due to the daily demands of caregiving, but also the psychological burdens—as our study finds many reports of caregivers feeling impaired by worry for the future, overwhelmed, or isolated. Our findings further suggest that providing care for a child with MLD could significantly diminish a caregiver’s emotional wellbeing. There is a clear need here to identify better personalized support services and counselling for caregivers that offers them the time and opportunities to focus on their own health and wellbeing.

To our knowledge, this study is the first to evaluate the professional and financial impact of caring for a child with MLD. Our results demonstrate that many caregivers sacrifice their career by either reducing employment or exiting the workforce altogether, which affects their potential for professional advancement and could put stress on the family’s finances given the forgone income. Financial support by means of government unemployment benefits, or donations from non-profit organizations are likely an important factor in minimizing the additional burden caused by forgone income.

### Differences based on time since diagnosis

As Kehrer, et al., 2011 described, the natural course of MLD typically starts with a period of developmental stagnation, followed by variable periods of plateaus and rapid disease progression, ultimately leading to a point of stabilization at a minimal functional level [22, 23]. This path was described by caregivers who participated in this study and is illustrated through the differences in caregiver-reported burden at different points in the individual’s disease progression. In assessing the effect of time since diagnosis on time investment, our study shows that caregivers of more recently diagnosed individuals with MLD make more frequent visits to receive medical care. This may be explained by the rapid rate of progression of MLD in its early stages and symptom stabilization overtime. We also see how MLD impacts families over time with directionally increasing reports of negative impact on familial relationship for caregivers who have been caring for their child with MLD for longer. As the disease stabilizes, our results show, directionally, caregivers who have been caring for a child who was diagnosed with MLD ≥ 6 years tend to have more satisfaction with their personal lives and are more able to keep up with family responsibilities and social commitments. Ammann-Schnell, et al., 2021 finds similar findings in their study of MLD and pontocerebellar hypoplasia type 2, where care for caregivers and families seems to be particularly important following diagnosis as well as during the diagnosed child’s end stage [18]. Nonetheless, the fact that these temporal differences in our study were not found statistically significant demonstrates the lasting impact of MLD that weighs on caregivers— similar to Amann-Schnell, et al.’s findings, we believe focusing family and caregiver support efforts early on in the diagnosis is critical, though ensuring those resources are still available throughout the family’s journey is still important to consider.

In the early years of the disease, caregivers may get closer to their spouses/partners while they navigate the new changes to their family. However, relationships become more negatively impacted as time goes on, as evidenced by our study. These findings align to reports from previous research, which found that some families with a child diagnosed with MLD do get closer [18].

### Differences by onset type

We know that individuals with late infantile MLD have a more rapid rate of functional decline compared to juvenile MLD [2, 5]. We also see these nuances in the nature of disease progression translate to some extent in the caregiver-reported burden of disease as well. Families who experience a child’s early loss of physical and cognitive function with a diagnosis of late infantile onset MLD are directionally more likely to report a negative impact on familial relationships. Given the early age of onset and rapid rate of progression, families often grapple with the shock, frustration, helplessness, and anxiety that can lead to serious stress on the marital relationship and potential dissolution as observed in a systemic review of couple’s relationships after the death of a child [24]. In the subgroup of caregivers who were caring for a child with juvenile onset MLD, we see directionally more caregivers reporting significant lifestyle changes and dissatisfaction with their personal lives. This difference could be in part a product of the nuances in childcare prior to the onset of symptoms— caregivers of individuals with late infantile MLD may have only had the experience of early childcare whereas in juvenile onset, the child may have developed some autonomy before the onset of regression and reverting back to the level and nature of their younger childhood needs. The dynamics of age at onset of symptoms and differences in progression of disease would be an interesting area to explore further in terms of long-term caregiver impact and family needs.

### Minimal differences in transplanted versus palliative care only subgroup analyses

In a few areas within this study, we explore the burden specifically for caregivers of individuals who were able to receive transplant (*n* = 6). Despite receiving transplant, individuals with MLD and their families still experienced a similar disease burden to those receiving supportive care only. Though directionally lower, there were no significant differences in number of inpatient and outpatient visits in the past year and for the actual transplant procedure many were forced to relocate from their homes, miss work, and make arrangements for their other children. These data may serve as a useful benchmark when evaluating new therapeutic options. The domains of impact used in this study could be used to understand the value of new treatments based on the extent of improvement in the holistic set of dimensions relevant to burden of disease compared to stem cell transplant and supportive care.

### Limitations

As MLD is an ultra-rare disease, this study is inherently limited by what typically would be considered a small sample size, which is accentuated when data are stratified. Although country-level data were presented, comparisons were not made across countries due to these limitations. However, based on the nature of the recruitment time and the epidemiology of the disease, the sample is deemed sufficient to make meaningful and insightful comparisons and this study contributes to the understanding of the challenges associated with caregiving for a child with MLD.

## Conclusions

This study captures the negative impact of MLD on caregivers’ lives broadly and across several key dimensions, including psychological and physical health, time investment, familial relationships, and career. We demonstrate how caregivers face significant challenges as their lives dramatically change after their child is diagnosed with MLD. Due to the debilitating nature of the disease, the day-to-day care activities require significant daily time commitment, consequently leading to diminished quality of life for caregivers. This is expressed through caregivers’ inability to handle daily activities to the same extent as pre-diagnosis, their lack of participation in social activities and the negative impact on their relations with others. In effect, caregivers often suffer mentally from anxiety and depression, and may even experience physical pain and discomfort from the labor-intensive care activities. Furthermore, their familial relationships, especially relationships with spouses or partners, are invariably impacted. In addition, they are often forced to reduce their participation in the workforce or to stop working all together, which in turn negatively impacts their family income. This study demonstrates that the impact of MLD on caregivers is multifaceted and highlights the need for caregiver and family assistance when a child is diagnosed with MLD. Beyond the impact on individual caregivers, this study underscores the resource implications of MLD to healthcare systems and society in general, e.g., through the high number of healthcare visits, the need nursing assistance, and decreased participation in the workforce. Overall, this study highlights the need for better therapeutic solutions and support for families impacted by MLD, and it is our hope that this study contributes to better patient care. Additionally, our findings may be applicable for other inherited lysosomal storage disorders, where research is limited.

### Supplementary Information

**Additional file 1.** Verbatims from qualitative interviews with study participants.

## Data Availability

The datasets generated and analyzed during this study are not publicly available due to individual patient privacy protections.

## Appendix survey structure and example questions

Metachromatic Leukodystrophy (MLD) Caregiver-Reported Quality of Life Assessment – Descriptive Table of Survey Sections and Questions.

Please reach out to Magnolia Innovation for additional details on the survey design.

**Table:**

life: Within the past	
**Section A: background**	
**Example Questions**	**Response type**
Which country are you based in?*	Single Select
What is your age? Please select from the appropriate range below.*	Single Select
For how many people with MLD are you, or have you been the primary caregiver?*	Numerical
For how long have you been the primary caregiver for this person?	Numerical
**Section B: pedsql general core scales**	
*Pediatric Quality of Life Inventory™ (PedsQL™) by Varni JW*^1^	
**Section C: symptom burden**	
Which of these answers comes closest to describing your child’s quality of life: At diagnosis?^2^	Scaler (1–5)
Which of these answers comes closest to describing your child’s quality of life: Within the past 4 weeks? ^2^	Scaler (1–5)
In the past 4 weeks, to what extent did any of the following physical symptoms impact your child as a result of their MLD: difficulty walking or crawling^6^	Scaler (1–5)
In the past 4 weeks, to what extent did any of the following physical symptoms impact your child as a result of their MLD: Breathing/ respiratory problems^6^	Scaler (1–5)
During the past 4 weeks, to what extent did your child’s condition interfere with his/her social activities with family, friends, neighbors, or groups?^3^	Scaler (1–5)
During the past 4 weeks, to what extent did your child’s condition interfere with his/her school attendance?	Scaler (1–5)
**Section D: treatment burden**	
Did your child’s disease progression cause them to be ineligible for a transplant?*	Yes/No
What kind of donor was the transplant done with?	Single Select
Was conditioning used in preparation for your child’s Stem Cell Transplant?	Yes/No
What kind of conditioning was used?	Open-end
**Section E: time investment**	
When you think back on the following time periods, how many times did you and your child go to the hospital (inpatient) for his/her MLD, and how many total days did you stay there? If you are unsure, your best estimate will do: Since Diagnosis	Numerical
When you think back on the following time periods, how many times did you and your child go to the hospital (inpatient) for his/her MLD, and how many total days did you stay there? If you are unsure, your best estimate will do: Within the Past 12 Months*	Numerical
When you think back on the following time periods, how many times did you and your child go to the hospital (inpatient) for his/her MLD, and how many total days did you stay there? If you are unsure, your best estimate will do: Within the Past 4 Weeks*	Numerical
Is this number of days in the past month a typical number of days spent at the hospital for you and your child?	Yes/No
What was the reason for the hospitalization(s) within the past 4 weeks?	Open-end
How many days on average did you stay at the hospital for each hospitalization?	Numerical
**Section F: social, emotional, and psychological burden**	
Has your social life changed since your child was diagnosed? Please indicate to what extent you agree with each of these statements: I am as active socially as I had been before my child was diagnosed*	Scaler (1–7)
Has your social life changed since your child was diagnosed? Please indicate to what extent you agree with each of these statements: I miss many of my leisure activities that I used to enjoy before my child was diagnosed*	Scaler (1–7)
These questions are about how you felt and how things were with you during the past 4 weeks. For each question, please give the one answer that comes closest to the way you were feeling. How much of the time during the past 4 weeks: Did you feel overwhelmed?^3^*	Scaler (1–6)
These questions are about how you felt and how things were with you during the past 4 weeks. For each question, please give the one answer that comes closest to the way you were feeling. How much of the time during the past 4 weeks: Did you feel calm and peaceful? ^3^*	Scaler (1–6)
During the past 4 weeks, to what extent did your emotional state interfere with your social activities with family, friends, neighbors, or groups?^3^	Scaler (1–5)
Within the past 4 weeks, in general, how would you rate your child’s mood?^2^	Scaler (1–5)
**Section G: financial and professional impact**	
During the past 4 weeks, did you have any of the following problems with your work or other regular daily activities: Cut down the amount of time you spent on work or other activities^3^*	Yes/No
During the past 4 weeks, did you have any of the following problems with your work or other regular daily activities: Experienced work problems/difficulties*	Yes/No
Did you or your spouse/partner have to miss work as a result of your child’s condition?*	Yes/No
When you think back, how many days did you and your spouse/partner have to miss work due to MLD? If you are unsure, your best estimate will do: Since Diagnosis	Numerical
When you think back, how many days did you and your spouse/partner have to miss work due to MLD? If you are unsure, your best estimate will do: Within the Past 12 Months	Numerical
When you think back, how many days did you and your spouse/partner have to miss work due to MLD? If you are unsure, your best estimate will do: Within the Past 4 Weeks*	Numerical
**Section H: demographics and closing**	
Which of the following best describes your relationship status?	Single Select
What is the highest level of education that you have attained?	Single Select
Through which of the following do you have your primary form of health insurance coverage?	Single Select
Which of the following best represents your annual family income (before taxes)?	Single Select

*Asterisks indicate questions included in manuscript

1. *Licensed:* Varni JW, Seid M, Kurtin PS. PedsQL 4.0: reliability and validity of the Pediatric Quality of Life Inventory version 4.0 generic core scales in healthy and patient populations. *Med Care*. 2001;39(8):800-812.

2.*Questions adapted from PROMIS:* Gruber-Baldini AL, Velozo C, Romero S, Shulman LM. Validation of the PROMIS^®^ measures of self-efficacy for managing chronic conditions. *Qual Life Res*. 2017;26(7):1915-1924.

3.*Questions adapted from SF-36:* Brazier JE, Harper R, Jones NM, et al. Validating the SF-36 health survey questionnaire: new outcome measure for primary care. *BMJ*. 1992;305(6846):160-164.

4.*Questions adapted from Neuro-QoL:* Salsman JM, Victorson D, Choi SW, et al. Development and validation of the positive affect and well-being scale for the neurology quality of life (Neuro-QOL) measurement system. *Qual Life Res*. 2013;22(9):2569-2580.

5.*Questions adapted from IMPA:* Brown TM, Martin S, Fehnel SE, Deal LS. Development of the Impact of Juvenile Metachromatic Leukodystrophy on Physical Activities scale. *J Patient Rep Outcomes*. 2017;2(1):15.

6.*Questions adapted from Eichler, et al. 2016:* Eichler FS, Cox TM, Crombez E, Dali CÍ, Kohlschütter A. Metachromatic Leukodystrophy: An Assessment of Disease Burden. *J Child Neurol*. 2016;31(13):1457-1463.
