# Supplementary material for: An international study of caregiver-reported burden and quality of life in metachromatic leukodystrophy
Source: Orphanet J Rare Dis. 2022 Sep 2;17:329. doi: 10.1186/s13023-022-02501-8 (PMC9438185; doi:10.1186/s13023-022-02501-8)
Supplement: Supplementary file 1 — Additional file 1. Verbatims from qualitative interviews with study participants. [file 13023_2022_2501_MOESM1_ESM.docx]

**An International Study of Caregiver-Reported** **Burden and Quality of Life in Metachromatic Leukodystrophy
*Supplemental Materials – Caregiver Quotes***

***Section 1. EQ-5D-5L***

- **Quote 1:** *“My biggest fear was losing who she was. We are grieving and we were grieving for the last nine years. It's an ongoing process, it never goes away. You see, any situation becomes the new normal, but it's a constant sadness that we deal with. It's not going to get better, but you've learned to live with it.”*- Caregiver (UK)
- **Quote 2:** *“I was crunched up in the corner and she had the whole bed. Before we had night cameras, I stayed up all night watching her and I think that put a strain on my health…taking care of myself has gone down the drain completely…it’s a parent’s fear to have a child with an illness, my biggest fear was losing her.”* - Caregiver (UK)
- **Quote 3:** *“Those transfers are the hardest physically. A day where she has 3-4 appointments, I got to transfer her so many times, plus you got a 90lb wheelchair that you got to load.”* –Caregiver (US)

***Section 2. Time Investment***

- **Quote 1:** *“I am her sole caretaker, but I do have her in therapies. So, speech once a week, occupational therapy once a week. Physical therapy is outside of the home and that's twice a week…and we have those appointments outside of the home that we need to kind of keep up with… ”* – Caregiver (US)
- **Quote 2:** *“There's not really any downtime. Maybe when she initially goes to bed and the other children are in bed, I might get an hour and a half, before in giving meds again and going through the whole process of getting up every couple of hours…t's not even sort of 7 hours, 12 hours, it's 24-hours a day.”* – Caregiver (US)

***Section 3. Familial Impact***

- **Quote 1:** *“We have 2 other children, they can get themselves ready and they do help with my son and sometimes it gets to me that an 8 y/o boy has to help his 13 y/o brother but it's just the way it is, my wife can't manage them all by herself, so everyone helps out.”* – Caregiver (US)

***Section 4: Social Impact***

- **Quote 1:** *“There’s small little times when we get away to do something, but as far as a social life, that’s gone. It’s sometimes hard to just go grocery shopping.”* – Caregiver (US)

***Section 5: Emotional Impact***

- **Quote 1:** *“I think the most challenging aspect has been not so much physical aspects…The most challenging aspect I think is—I'm going to tear up here—is really feeling, dealing with caring for him while you know he's going to die and there's nothing you can do, watching him dying in slow motion. So that's definitely the hardest thing to deal with.”* – Caregiver (US)

***Section 6: Professional Impact***

- **Quote 1:** *“I tried going back to work this year, but I wasn’t able to. I just left. Told them I couldn’t do it…we met our out-of-pocket max for the whole family in January, that was the hardest because the hospitalizations and all of that was on us.” – Caregiver ((US)*
